# Snapshot of Examination Usage in Emergency Medicine Clerkships

**DOI:** 10.7759/cureus.67301

**Published:** 2024-08-20

**Authors:** William D Alley, Iltifat Husain, Blake Briggs, David Story

**Affiliations:** 1 Department of Emergency Medicine, Wake Forest School of Medicine, Winston-Salem, USA; 2 Department of Emergency Medicine, University of Tennessee Medical Center, Knoxville, USA

**Keywords:** medical student clerkship, end-of-clerkship exam, multiple-choice questions, assessment in health professions education, undergraduate medical education

## Abstract

Objective

Emergency Medicine (EM) clerkships often use a written exam to assess the knowledge gained over the course of an EM rotation in medical school. Clerkship Directors (CDs) may choose the National Board of Medical Examiners (NBME) EM Advanced Clinical Science Subject Exam (ACE), the Society for Academic Emergency Medicine (SAEM) M4 exam, which has two versions, the SAEM M3 exam, or departmental exams. There are currently no published guidelines or consensus regarding their utility. This survey-based study was designed to collect data regarding current practices of EM clerkship exam usage to analyze trends and variability in what exams are used and how.

Methods

The authors designed a cross-sectional observational survey to collect data from EM CDs on exam utilization in clerkships. The survey population consisted of clerkship directors, assistant clerkship directors, or faculty familiar assessments in their EM clerkship. Initial dissemination was by electronic distribution to subscribers of the Clerkship Directors in Emergency Medicine (CDEM) list-serve on the SAEM website. Subsequently, contact information of CD’s from institutions that had not responded was obtained by manual search of the Emergency Medicine Residents’ Association (EMRA) Match website and individual correspondence was sent at regular intervals. Data obtained include clerkship characteristics, exam used, weight of the exam relative to the overall grade, and alternatives if the preferred exam was previously taken.

Results

Eighty-seven programs (42% response rate) completed the survey between August 2019 and February 2021. Of the 87 responses, 71 (82%) were completed by a CD. Forty-six (53%) institutions required an EM rotation. Students were tested in 34 (74%) required EM clerkships and 48 (69%) out of 70 EM electives. In required rotations that used an exam, 20 (59%) used the NBME EM ACE, while 28 of 46 (61%) of EM electives that reported an exam used the SAEM M4 Exam. Five (15%) of the required clerkships used a departmental exam. Of clerkships requiring an exam, 46 (57%) weighed the score at 11-30% of the final grade. Data for extramural rotations mirrored that of EM electives. One-third of respondents indicated they do not inquire about previously taken exams.

Conclusion

This survey demonstrates significant variability in the type of exam, the weighting of the score, and alternatives if the preferred exam was previously taken. The lack of a consistent approach in how these exams are used in determining students’ final EM grades diminishes the reliability of the EM clerkship grade as a factor used by residency directors in choosing future residents. Further research on optimal usage of these exams is needed.

## Introduction

Across the United States, Emergency Medicine (EM) clerkships have been established to expose both third- and fourth-year medical students to the field of EM [1-3]. Knowledge-based exams are a core component of these clerkships. Multiple choice exams are often administered at the end of these clerkships to assess medical knowledge in EM [4,5]. Most of these exams are standardized and available to clerkship directors, including the National Board of Medical Examiners (NBME) Emergency Medicine Advanced Clinical Science Subject Exam (ACE) or the Society of Academic Emergency Medicine (SAEM) exam. The SAEM exam has three versions, EM-M4 versions 1 and 2 written for fourth-year students, and EM-M3, written for third-year students [6,7]. With a multitude of exams available, there is a paucity of literature showing what types are most being used.

Many students complete multiple EM clerkships in preparation for application into residency programs [8]. It has previously been documented that 59% of clerkship directors use a final, written exam, however this survey was performed in 2010 and before the ACE was created [6]. Due to a lack of consensus on the types of exams to use in EM clerkships, students may take the same exam in different clerkship locations. To our knowledge, there are no studies that evaluate the current exam structure on EM clerkships across the United States, as well as the implementation strategy in cases where students may have already completed the available exams.

In this study, we examine how clerkship directors (CDs) administer knowledge-based exams at the end of EM clerkships in order to identify trends and variability. Secondly, we look at what strategies are utilized when students have already taken a clerkship’s preferred exam. We believe these are important questions as these exams play a significant role in the overall evaluation of medical students, and variability in how these exams are used could insert a degree of unreliability in clerkship grades that are a factor used by residency programs in evaluating potential residency candidates, advantaging some while disadvantaging others. This variability could call into question the importance residency program directors place on the EM clerkship grade, potentially shifting it to other factors when evaluating residency applicants. At the same time, clerkship directors may use this data to spur a movement toward national consensus on exam usage to provide more consistency in the evaluation of medical students across institutions.

The abstract for this study was previously presented as a poster presentation at the 2023 CORD Academic Assembly in Las Vegas, Nevada on March 22, 2023.

## Materials and methods

This was a cross-sectional observational survey targeting EM CDs at medical schools accredited by the Liaison Committee on Medical Education (LCME). EM CDs were identified by contact information available on the Emergency Medicine Residents’ Association (EMRA) Match website (https://webapps.emra.org/utils/spa/match#/search/map). Data was collected from surveys completed between February 2019 and August 2021. The study met criteria for exemption from informed consent by the Institutional Review Board of the Wake Forest School of Medicine (approval IRB00069527).

This survey was developed and disseminated based on best-practice principles described by Hoddinott et al. [9]. Survey items were first derived based on consensus of expert educators, including residency and clerkship leadership and the authors. Items were selected based on their relation to the use of exams as part of EM clerkship grading, including characteristics of the EM clerkship, whether an end-of-clerkship exam is used, relative weight of the exam grade in relation to the overall grade, and specific strategies used if a student has previously completed the preferred exam used by a clerkship. After review of preliminary items, comments on item comprehensibility, survey comprehensiveness, and content validity were obtained from independent EM medical student educators. Survey revisions were made based on this information, and the refined survey was then piloted with a small group of EM educators and clerkship directors. Adjustments were made based on respondent feedback and the survey finalized by consensus opinion. A complete copy of the survey is available online (https://www.surveymonkey.com/r/F2LWCLX).

Initial survey dissemination was conducted using an online survey service (SurveyMonkey,™ Palo Alto, CA, USA). First, a survey link was delivered to CDs over the Council of Residency Directors in Emergency Medicine (CORD) listserv. This community consists of 342 members. A second electronic correspondence was sent nine days later. Non-responders were identified and personalized emails containing a survey link were sent to clerkship directors at the email address listed on the EMRA Match website list of EM clerkships when accessed in April-June 2019. Responses were then analyzed for completion and program specificity. Duplicate responses from the same institution were evaluated and responses were prioritized as follows: clerkship director, assistant clerkship director, residency director, assistant residency director, other. Descriptive statistics were calculated and are reported.

## Results

Ninety individuals completed the survey. Three institutions submitted two responses and duplicates were removed from the analysis. Two additional institutions submitted initially incomplete surveys that were subsequently completed in full. Data presented is based on the 87 responses out of an estimated total of 207 institutions contacted based on the number of clerkships listed on the EMRA Match website at the time of survey dissemination, resulting in a response rate of 42%. It should be noted that an accurate number of active EM clerkships is elusive due to variability from year to year, and a clerkship listing may remain despite inactivity. In order to evaluate for generalizability of the responses, an assessment of the regionality of respondents was performed. There were at least nine institutions responding from each U.S. region listed below, in addition to three international institutions. 

**Figure 1:**
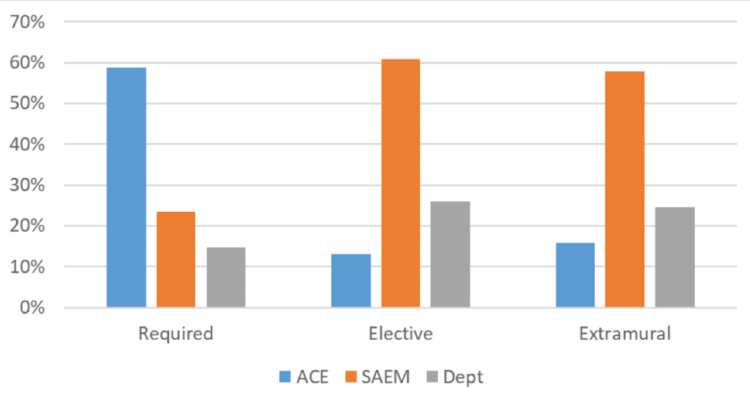
Exam Offerings by Emergency Medicine Clerkship Type Exam Offerings by Emergency Medicine Clerkship Type (ACE, NBME Advanced Content Exam in Emergency Medicine; SAEM: Society of Academic Emergency Medicine M4 Exam, Dept: exams created by the clerkship faculty)

CDs or assistant CDs completed 75 (86.2%) of the surveys, residency leadership five (5.7%) of the surveys, and the remaining seven (8.1%) of surveys were completed by someone in another role familiar with clerkship grading. Over half of respondents (46/87) reported that EM is a required rotation at their institution. Within the subset of programs with required EM rotations, 12 (26.1%) take place in the third year, 24 (52.2%) in the fourth year, and 10 (21.7%) in either the third or fourth years. Thirty-four (73.9%) institutions with required clerkships have their students take a written exam at the end of the rotation, with the ACE being the most commonly used in 20 (58.8%) of them. An SAEM exam (EM-M4 version 1, EM-M4 version 2 or EM-M3) is offered at eight (23.5%) and a departmental exam written by the home institution is offered at five (14.7%) (Figure 1). A single respondent was unable to specify the exam used. Most clerkships, 21 out of 34 (61.8%), weigh the exam at 11-30% of the final grade, six (17.7%) weigh it <10% of the final grade, five (14.7%) at 31-50% of the final grade, and two (5.9%) weigh it > 50% of the final grade.

A great majority of institutions surveyed, 70/87 (80.5%), offer an elective in EM, with 48 (68.6%) of those programs administering an end-of-rotation exam. Most clerkships, 28 out of 46 (60.9%) who reported, use an SAEM exam at the end of the elective with 12 (26.1%) administering a departmental written exam and six (13.0%) utilizing ACE (Figure 1). Just over half of institutions, 25 out of 46 (54.4%), weigh that exam between 11-30% of the final grade, 18 (39.1%) at < 10% of the final grade and three (6.5%) between 31-50%. No elective exams constituted greater than 50% of the final grade calculation. 

Extramural EM electives are available at 91.9% (79/86) of respondents’ institutions, with an end-of-rotation exam being a requirement at 58 (73.4%) of those. Nearly 60%, 34 out of 57 (57.9%), reported using a version of the SAEM exam, and nearly a quarter, 14 of 57 (24.6%), use departmental exams. Most of the remaining clerkships, nine (15.8%), use the ACE (Figure 1) with a single clerkship using an Objective Structured Clinical Exam (OSCE). The end-of-rotation exams are mostly weighted at either 0-10% of the final grade (20 of 57, 35.1%) or 11-30% of the final grade (33 of 57, 57.9%), with a much smaller proportion weighing 31-50% (3 of 57, 5.3%) or >50% (1 of 57, 1.8%). 

**Figure 2:**
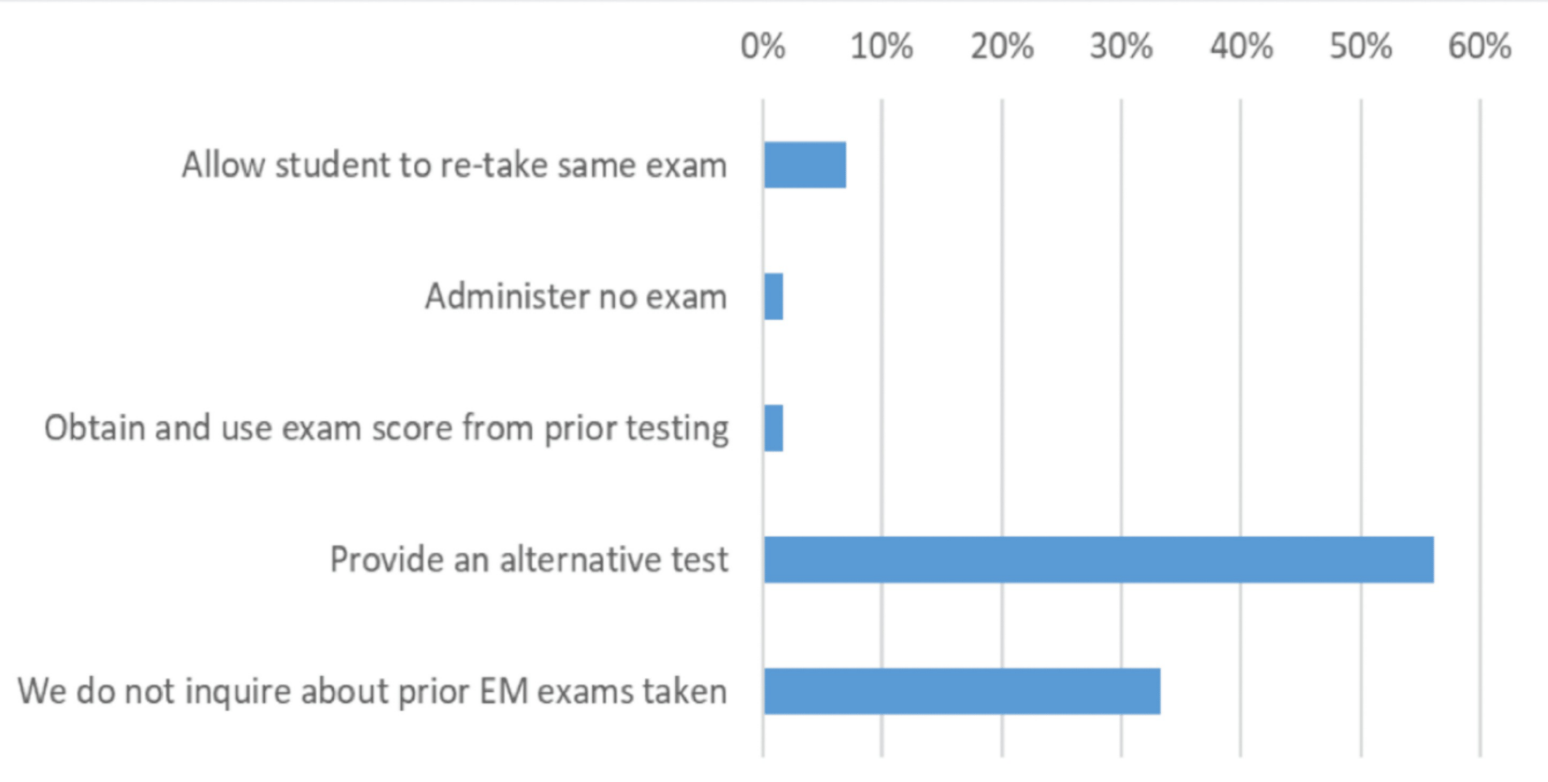
Responses to “What is your policy if an extramural (visiting) student has previously taken the given exam during a prior EM rotation?” EM: Emergency Medicine

The 57 clerkships that provide an end-of-rotation exam to extramural students were surveyed about practices for administering alternative exams if the student has already taken the exam offered. The majority of institutions, 32 (56.1%), provide an alternative exam, while one-third (19 of 57, 33.3%) do not inquire about prior tests taken, and four (7.0%) allow the student to retake an exam (Figure 2). 

## Discussion

The use of end-of-clerkship exams to assess student performance is commonplace among US medical schools. This is consistent with prior literature findings [7]. However, our findings show variability in how these multiple-choice exams are used in both the test administered and their weighting in final rotation grade calculation. Additionally, more than a third of programs do not ask extramural students if they have taken a similar multiple-choice exam before.

Previous literature suggests that EM program directors place significant emphasis on student performance during EM clerkships in their selection criteria [8,10]. Our findings demonstrate that written exams are a major grading component contributing to clerkship grades. However, due to the variability in how these tests are used, EM program directors could be making comparisons among medical students based on unequal metrics, advantaging some and disadvantaging others. For example, a student who historically tests well is likely to earn a higher grade at a program that heavily weighs an exam score, while a student at an institution that weighs the exam minimally does not have the same opportunity to leverage an excellent test score to bolster their overall grade. 

Multiple organizations recommend applicants to EM residency complete a total of two core EM clerkships, with allowances for individual circumstances that may benefit the student to complete three EM clerkships [11]. With each additional clerkship, our data suggests that a student is increasingly likely to be assessed with the same exam used in a previous clerkship. There is conflicting evidence on the effect of repeated exposure to questions on exams, but more robust scholarship in this area is needed to ensure the validity of the exam [12,13].

It is interesting to note that required clerkships are far more likely to use the ACE exam, while elective and extramural clerkships more commonly use SAEM exams or departmental exams. It is beyond the scope of this study to directly address the reasons for this, but some inferences can be cautiously made. First, NBME exams are commonly utilized in required clerkships for other specialties, making the natural consistency of using the ACE for EM attractive [14-16]. Second, the NBME has developed a reputation for its assessments across multiple specialties and levels of training, giving the impression of reliability and validity. Finally, cost may be a significant concern. At approximately 50 dollars per exam administration, offering the ACE comes with a large expense compared to the SAEM or departmental exams, which are offered at no additional cost.

Given these findings, it is apparent that the assessment of the knowledge base of students on EM clerkships varies amongst institutions, sometimes widely. This has implications for multiple stakeholders. First, residency program directors should be aware of this variability when using the clerkship grade as a major factor in evaluating potential residents. Clerkship directors should be clear about the metrics that are used to assign an overall clerkship grade and how they are used in order to provide a clearer assessment of a student’s clerkship performance.

This is a voluntary survey subject to selection and recall bias, and includes data from under half of the clerkships surveyed. However, the 42% response rate is consistent with the weighted mean response rate of online surveys in education-related fields, based on a meta-analysis by Wu et al. [17]. It also includes responses from a broad geographical distribution. Given the wide geographical distribution of respondents, we believe the data is at least reasonably representative of the whole.

## Conclusions

We have found significant variability between EM clerkships in regards to the type of multiple choice exams used, the weighting of exam scores in relation to final grades, and the strategies used if a student has previously completed the preferred exam used by a clerkship. As end-of-clerkship exams are often used as a component of the overall clerkship grade, residency program directors should be aware of this variability when using the clerkship grade as a major factor in evaluating potential residents. Clerkship directors, too, should be aware of this variability as they consider how their approach to assessment presents an overall evaluation of a student compared to others within a residency interview pool.

## Appendices

Cover Letter:

We are conducting a survey about testing practices in EM rotations to assess whether the available examinations are sufficient given the increased number of schools with required EM rotations, continued elective EM clerkships, and the proclivity for students to do multiple away EM rotations. Your participation would be much appreciated. The survey below should not take longer than 3 minutes to complete.

Survey on Student Testing Practices in Emergency Medicine Rotations

1. Name of your institution?

2. What is your role?

  Clerkship Director

  Assistant Clerkship Director

  Residency Program Director

  Assistant Residency Program Director

  Other

3. Is emergency medicine (EM) a required rotation at your institution?

  Yes

  No

4. When do your students take the required EM course?

  3rd year

  4th year

  either

5. Do students take an end-of-rotation examination during the required EM course?

  Yes

  No

6. What test is preferentially administered during the required EM rotation?

  Advanced Clinical Examination in Emergency Medicine (NBME)

  National Emergency Medicine M4 Exam, version 1 (SAEM)

  National Emergency Medicine M4 Exam, version 2 (SAEM)

  Departmental exam (written by home institution)

  Other (please specify)

7. How much weight does the examination carry in the required EM rotation?

  0-10%

  11-30%

  31-50%

  >50%

8. Does your institution offer an EM elective?

  Yes

  No

  Only subspecialties (i.e. Pediatric EM, Toxicology, Ultrasound, EMS, etc)

9. Is an end-of-rotation examination administered during the EM elective?

  Yes

  No

10. What test is administered to students in the EM elective?

  Advanced Clinical Examination in Emergency Medicine (NBME)

  National Emergency Medicine M4 Exam, version 1 (SAEM)

  National Emergency Medicine M4 Exam, version 2 (SAEM)

  Departmental exam (written by home institution)

  Other (please specify)

11. How much weight does the examination carry in the elective EM rotation?

  0-10%

  11-30%

  31-50%

  >50%

12. Do you offer an extramural (visiting) EM rotation at your institution?

  Yes

  No

13. Do students take an end-of-rotation examination during the extramural (visiting) EM elective?

  Yes

  No

14. What test is administered to students in the extramural (visiting) EM course?

  Advanced Clinical Examination in Emergency Medicine (NBME)

  National Emergency Medicine M4 Exam, version 1 (SAEM)

  National Emergency Medicine M4 Exam, version 2 (SAEM)

  Departmental exam (written by home institution)

  Other (please specify)

15. How much weight does the examination carry in the extramural (visiting) EM rotation?

  0-10%

  11-30%

  31-50%

  >50%

16. What is your policy if an extramural (visiting) student has previously taken the given exam during a prior EM rotation?

  Allow the student to re-take the same examination

  Administer no examination

  Obtain and use the examination score the student received with prior testing

  Provide an alternative test

  We do not inquire about EM exams taken during previous rotations

17. What alternative test is used in this case?

  Advanced Clinical Examination in Emergency Medicine (NBME)

  National Emergency Medicine M4 version 1 (SAEM)

  National Emergency Medicine M4 version 2 (SAEM)

  Departmental exam (written by home institution)

  Other (please specify)
